# Tongxinluo is associated with neurovascular changes following rt-PA-treated ischemic stroke: associations with inflammation, vascular remodeling, and neural plasticity

**DOI:** 10.3389/fneur.2026.1861033

**Published:** 2026-10-14

**Authors:** Meiling Xiao, Wei Ding, Xiaoqu Zuo, Hongwu Tao

**Affiliations:** 1 Second Clinical College, Liaoning University of Traditional Chinese Medicine, Shenyang, Liaoning, China; 2 Department of Rehabilitation Medicine, Central Hospital Affiliated to Shenyang Medical College, Shenyang, Liaoning, China

**Keywords:** acute ischemic stroke, angiogenesis, neuroinflammation, neurovascular unit remodeling, rt-PA, synaptic plasticity, Tongxinluo

## Abstract

**Background:**

Recombinant tissue plasminogen activator (rt-PA) is the standard treatment for acute ischemic stroke but is often limited by microvascular obstruction and reperfusion injury. Tongxinluo (TXL) has demonstrated neuroprotective potential, yet its association with neurovascular responses during rt-PA-associated reperfusion remains incompletely characterized.

**Methods:**

Because reperfusion was achieved by filament withdrawal before rt-PA administration, this model evaluates TXL-associated effects during mechanically induced reperfusion with rt-PA exposure rather than enhancement of rt-PA-mediated thrombolysis. Animals were randomized into Sham, MCAO, MCAO + rt-PA, and MCAO + rt-PA + TXL (low/high dose) groups. Neurological outcomes were assessed longitudinally. Cerebral perfusion was monitored using laser speckle contrast imaging (LSCI). Mechanistic analyses included assessments of blood-brain barrier (BBB) integrity, oxidative stress, microglial activation, angiogenesis, mitochondrial function, apoptosis, and synaptic plasticity markers (BDNF, GAP-43, and Synapsin I) using histological, biochemical, and molecular techniques.

**Results:**

Adjunctive TXL treatment was associated with improved neurological outcomes and reduced infarct volume compared with rt-PA alone, with dose-dependent trends observed. TXL was associated with improved cortical microvascular perfusion and reduced severity of the no-reflow phenomenon. Mechanistically, TXL suppressed microglia-mediated neuroinflammation (downregulating TNF-α, IL-1β, and IL-6) and preserved BBB integrity. This anti-inflammatory environment facilitated angiogenic maturation, evidenced by increased microvessel density and lumen formation. Furthermore, TXL stabilized mitochondrial function, inhibited neuronal apoptosis, and upregulated BDNF, GAP-43, and Synapsin I, leading to significant restoration of dendritic spine density.

**Conclusion:**

TXL may provide adjunctive neuroprotective effects in the context of rt-PA treatment by modulating inflammatory, vascular-associated, and neuronal recovery responses. These findings support further investigation of TXL as a complementary therapy following reperfusion. By concurrently suppressing neuroinflammation and promoting vascular and synaptic reconstruction, TXL translates multi-target modulation into robust functional recovery, supporting its potential as a strategic combination therapy for acute ischemic stroke.

## 1 Introduction

Despite advances in acute reperfusion strategies, ischemic stroke continues to represent a paramount challenge in global health, characterized by high rates of mortality and persistent neurological disability (1). Recombinant tissue plasminogen activator (rt-PA) remains the cornerstone of pharmacological intervention, exerting its therapeutic effect by dissolving occlusive thrombi to restore macrovascular blood flow (2, 3). However, clinical outcomes following rt-PA administration are often suboptimal. A significant proportion of patients experience incomplete tissue reperfusion due to the “no-reflow” phenomenon at the microvascular level, alongside severe secondary injuries driven by ischemia-reperfusion (I/R) cascades (4). These cascades include oxidative stress exacerbation, blood-brain barrier (BBB) breakdown, and a robust inflammatory response that collectively undermine the potential benefits of thrombolysis (5–7). However, experimental models remarkably differ in their ability to reproduce thrombus-dependent fibrinolysis. In filament-based MCAO models, reperfusion is typically achieved mechanically rather than through rt-PA-mediated thrombus dissolution. Consequently, developing adjunctive therapies that not only extend the therapeutic window of rt-PA but also actively protect the cerebral microenvironment from I/R-induced damage is a critical priority in stroke research.

Central to the pathophysiology of stroke-induced injury is the disruption of the neurovascular unit (NVU), a functional entity comprising endothelial cells, pericytes, astrocytes, microglia, and neurons (8). Under ischemic conditions, the NVU transitions from a homeostatic state to a cytotoxic milieu. Resident microglia rapidly activate, adopting an amoeboid morphology and releasing pro-inflammatory mediators, such as TNF-α, IL-1β, and IL-6 (9–12). This inflammatory surge not only directly damages neuronal structures but also impairs endothelial integrity, thereby hindering effective angiogenesis and vascular remodeling in the ischemic penumbra. Conversely, a failure to re-establish a mature vascular network perpetuates hypoperfusion and limits the delivery of neurotrophic support necessary for synaptic reconstruction (13–15). Therefore, therapeutic interventions capable of simultaneously dampening microglial-mediated inflammation and fostering structural vascular maturation may offer a synergistic approach to preserving NVU integrity and enhancing functional recovery post-thrombolysis.

Tongxinluo (TXL), a patented traditional Chinese medicine formulation, has garnered attention for its multi-target protective effects in cardiovascular and cerebrovascular diseases. Comprising a complex matrix of herbal extracts, TXL has been shown to exhibit anti-inflammatory, antioxidant, and endothelium-stabilizing properties in various experimental models (16, 17). Although TXL has been reported to exert antioxidant and cytoprotective effects in ischemic injury (18), the molecular signaling pathways responsible for these effects in the setting of reperfusion remain incompletely defined. In particular, it remains unclear whether the TXL-associated enhancement of endogenous antioxidant defenses involves activation of canonical redox-responsive pathways such as the nuclear factor erythroid 2-related factor 2 (Nrf2)/heme oxygenase-1 (HO-1) signaling axis. Accordingly, the present study evaluated biochemical indices of oxidative injury and antioxidant capacity, while recognizing that upstream pathway activation requires direct molecular validation. While previous studies have demonstrated protective effects of TXL in experimental cerebral ischemia, its influence on reperfusion-associated tissue injury and neurological recovery in the context of rt-PA treatment remains insufficiently characterized. Beyond experimental studies, emerging clinical evidence has suggested potential neurological benefit of TXL in patients with acute ischemic stroke. In the multicenter randomized TISS trial involving 2,007 patients with acute ischemic stroke, adjunctive TXL treatment was associated with a higher probability of favorable functional outcome at 90 days compared with placebo (16). However, this trial was not specifically designed to evaluate the interaction between TXL and intravenous thrombolysis, and the authors noted that the prespecified subgroup analyses had limited statistical power, particularly among patients receiving reperfusion therapy, precluding definitive conclusions regarding thrombolysis-treated patients (16). More recently, a retrospective clinical study of 164 patients with acute cerebral infarction directly compared alteplase thrombolysis alone with TXL combined with alteplase and reported a higher overall clinical response rate together with favorable changes in cerebrovascular function, platelet activity, neuronal injury-related biomarkers, vascular endothelial function, and oxidative-stress indices in the combination group, without a significant difference in the overall incidence of reported adverse events (19). Nevertheless, the retrospective design, relatively limited sample size, and lack of rigorous prospective evaluation of thrombolysis-specific efficacy and hemorrhage-specific safety preclude definitive conclusions regarding the clinical benefit–risk profile of TXL combined with rt-PA (19). Thus, although preliminary human evidence supports further investigation of this therapeutic combination, its biological effects in the setting of reperfusion and the mechanisms underlying potential additional benefit remain incompletely defined (16, 19). Therefore, the present study was designed primarily to determine whether TXL could attenuate post-reperfusion injury and improve neurovascular and neurological recovery in rt-PA-treated ischemic stroke.

Against this background of emerging but still limited clinical evidence, the present study employed a transient middle cerebral artery occlusion (MCAO) model to characterize the biological effects associated with TXL administration in rt-PA-treated ischemia/reperfusion injury. Particular emphasis was placed on cortical tissue perfusion, inflammatory responses, vascular-associated remodeling, oxidative injury, mitochondrial membrane potential (MMP), and indices of neuronal plasticity. We hypothesized that TXL administration in rt-PA-treated MCAO rats would provide additional neuroprotective benefit and would be associated with favorable changes in inflammatory, vascular, mitochondrial, and neural plasticity-related responses. Because the experimental design did not include an MCAO + TXL-alone group, the present study was not designed to determine whether these effects reflected a specific additive or synergistic interaction between TXL and rt-PA or the independent neuroprotective activity of TXL. Specifically, because rt-PA was administered after mechanical reperfusion, the present study was designed to investigate TXL-associated effects in an rt-PA-treated reperfusion environment rather than to evaluate enhancement of rt-PA thrombolytic activity. Using laser speckle contrast imaging (LSCI) to characterize cortical perfusion dynamics, together with histological, biochemical, and molecular assessments, we examined whether these NVU-related changes accompanied the functional benefits of combined TXL and rt-PA treatment. Because the present experimental design did not directly manipulate individual cellular or molecular pathways, these analyses were intended to identify associated biological responses rather than establish causal relationships among the different components of NVU recovery.

## 2 Materials and methods

### 2.1 Animals and ethical statement

Adult male Sprague-Dawley (SD) rats, which aged 8–10 weeks, were purchased from Wukong Biological Technology Co., Ltd. (China). Male rats were selected to reduce biological variability associated with fluctuations in ovarian hormones, particularly estrogen, which can influence ischemic brain injury, neuroinflammation, blood–brain barrier integrity, and responses to cerebral ischemia/reperfusion. However, because only male animals were included, potential sex-dependent differences in ischemic injury, neuroinflammatory responses, blood–brain barrier disruption, and treatment responsiveness were not evaluated. All animals were housed in a specific pathogen-free (SPF) facility under controlled environmental conditions, maintaining a temperature of 22 °C ± 2 °C and humidity of 50% ± 10% with a 12-h light/dark cycle. Standard chow and water were provided *ad libitum*. All experimental procedures were conducted in strict accordance with the Guide for the Care and Use of Laboratory Animals published by the US National Institutes of Health and were approved by the Ethic Committee of Shenyang Medical University of TCM (Approval No.: SYYXY2025091105).

### 2.2 Experimental design and drug administration

A total of 65 rats were initially enrolled and randomly allocated in equal numbers (*n* = 13 per group) to five experimental groups: Sham, MCAO, MCAO + rt-PA, MCAO + rt-PA + TXL-Low, and MCAO + rt-PA + TXL-High. Randomization was performed using a computer-generated random-number sequence before surgical intervention. Investigators responsible for neurological assessment, histological quantification, and biochemical analyses were blinded to group allocation. The Sham group underwent surgical exposure of the cervical vessels without occlusion. The MCAO group served as the disease control and received intragastric normal saline following model induction. The MCAO + rt-PA group received an intravenous injection of rt-PA (10 mg/kg) via the tail vein immediately after reperfusion. The two treatment groups received the same dose of rt-PA followed by once-daily intragastric administration of TXL at 0.3 g/kg (low dose) or 0.6 g/kg (high dose) for 14 consecutive days after reperfusion. The selected dose range was based on previously established experimental use of TXL in rodent models of cardiovascular and cerebrovascular injury. TXL doses of 0.3 and 0.6 g/kg have been directly evaluated in experimental cardiovascular injury models, with 0.3 g/kg representing a lower pharmacologically active exposure and 0.6 g/kg providing a two-fold higher dose for assessment of dose-dependent effects (20, 21). In addition, intragastric TXL administration for up to 14 days after focal cerebral ischemia has been shown to promote neurovascular remodeling and functional recovery in rats (22). Dose-ranging studies in cerebral ischemia/reperfusion models have also demonstrated neuroprotective effects across TXL doses extending from 0.4 to 1.6 g/kg/day, supporting the use of 0.3–0.6 g/kg within the lower range of previously employed experimental exposures (23). Accordingly, 0.3 and 0.6 g/kg were selected to remain within a previously used preclinical dose range while permitting evaluation of whether increasing TXL exposure was associated with greater neuroprotective efficacy. Control groups received equivalent volumes of distilled water or saline. The required sample size was estimated a priori using G^*^Power (version 3.1.9.7) for one-way analysis of variance among five experimental groups. Assuming a large standardized effect size (*f* = 0.50), a two-sided α level of 0.05, and 80% statistical power, the analysis indicated that at least 53 evaluable animals were required. To compensate for an anticipated attrition rate of approximately 15% resulting from unsuccessful MCAO induction, perioperative mortality, or technical loss of samples, 65 animals (13 per group) were initially enrolled. The present experimental design did not include an MCAO + TXL-alone group. Accordingly, the study was designed to evaluate whether TXL provided additional benefit when administered in the context of rt-PA treatment, but it was not designed to distinguish an independent neuroprotective effect of TXL from a specific additive or synergistic interaction between TXL and rt-PA. Because the Sham and MCAO procedures are surgically distinguishable, the investigator performing the operation could not be blinded to whether cerebral artery occlusion was performed; however, the surgeon remained blinded to the subsequent treatment allocation among MCAO animals at the time of model induction. Treatment codes were assigned independently, and personnel involved in outcome assessment were not involved in treatment allocation or administration. Neurological behavioral testing, LSCI analysis, TTC infarct-volume quantification, Evans Blue extravasation measurement, immunofluorescence and immunohistochemical quantification, Western blot densitometry, TUNEL analysis, JC-1 fluorescence quantification, and Golgi-Cox dendritic spine analysis were performed using coded animal or sample identifiers by investigators unaware of treatment-group allocation. Where image-based analyses were performed, images were coded before quantification so that the assessor could not identify the corresponding experimental group. Group codes were revealed only after the prespecified measurements and primary statistical analyses had been completed.

### 2.3 Experimental timeline and tissue collection

Following 2 h of MCAO, the intraluminal filament was withdrawn to initiate reperfusion, and rt-PA was administered immediately thereafter to the designated treatment groups. TXL administration was initiated following reperfusion and continued once daily for 14 consecutive days. Neurological function was evaluated longitudinally at 1, 3, 7, and 14 days after reperfusion using the modified Neurological Severity Score (mNSS), Corner Turn Test, and Cylinder Test.

To distinguish acute ischemia/reperfusion injury from subsequent neurovascular and neuronal remodeling, animals were allocated to prespecified acute and recovery-phase assessment cohorts. In the acute-injury cohort, animals were euthanized at 24 h after reperfusion. Infarct volume and blood–brain barrier permeability were evaluated using TTC staining and Evans Blue extravasation, respectively. Ischemic penumbra tissues collected at the same 24-h time point were used for ELISA measurement of TNF-α, IL-1β, and IL-6, determination of oxidative-stress indices including MDA, SOD, and GPx, TUNEL assessment of apoptotic cell death, and JC-1 evaluation of MMP. These endpoints were selected to characterize the early inflammatory, oxidative, apoptotic, and mitochondrial responses associated with acute reperfusion injury.

A separate recovery-phase cohort was followed for 14 days after reperfusion and underwent serial neurological assessment throughout the observation period. At day 14, animals were euthanized and brain tissues were collected for evaluation of neurovascular remodeling and neural plasticity. Iba-1 and CD31 immunofluorescence were performed to assess persistent microglial activation and microvascular-associated changes, respectively, whereas CD34 immunohistochemistry was used to characterize vascular-associated lumen-like structures. Ischemic penumbra tissues collected at day 14 were also subjected to Western blot analysis of VEGF, BDNF, GAP-43, and Synapsin I to evaluate molecular changes associated with vascular repair and neuronal plasticity. In parallel, Golgi-Cox staining was performed on freshly collected brain tissue to quantify dendritic spine density as a structural index of neuronal plasticity. Because TTC staining, Evans Blue analysis, biochemical assays, and tissue-based histological or molecular assessments required terminal tissue collection or incompatible tissue-processing procedures, separate animals were assigned to the corresponding endpoint cohorts.

### 2.4 Laser speckle contrast imaging and cerebral perfusion analysis

Cortical cerebral blood flow was dynamically evaluated using laser speckle contrast imaging (LSCI) to characterize ischemia-induced hypoperfusion and the subsequent recovery of tissue perfusion following reperfusion. During imaging, rats were maintained under inhalational isoflurane anesthesia (approximately 1.5%−2.0% in oxygen), and body temperature was maintained at 37.0 °C ± 0.5 °C using a thermostatically controlled heating pad. Animals were positioned in a stereotaxic frame to minimize motion-related artifacts throughout image acquisition.

Following a midline scalp incision, the dorsal skull surface was exposed and gently cleaned with sterile physiological saline. The intact skull was used as the imaging window, and the surface was maintained moist throughout image acquisition to minimize optical artifacts. Particular care was taken to avoid mechanical damage to the skull or underlying cortical vasculature. The LSCI camera was positioned perpendicular to the skull, and the field of view encompassed the cortical territories supplied by both middle cerebral arteries. Camera position, imaging distance, exposure parameters, field of view, illumination intensity, and color-scale settings were maintained constant for all animals and experimental time points.

LSCI measurements were obtained before MCAO induction to establish baseline cortical perfusion, at the end of the 2-h ischemic period immediately before filament withdrawal, during the early reperfusion period following mechanical recanalization, and at 24 h after reperfusion. For early reperfusion assessment, recordings were acquired after cerebral blood flow had reached a stable post-withdrawal level. Representative perfusion maps were generated for visualization; however, all statistical comparisons were based on quantitative perfusion measurements rather than qualitative interpretation of pseudocolor images.

For quantitative analysis, anatomically matched regions of interest (ROIs) were predefined within the ipsilateral cortical territory supplied by the middle cerebral artery and within the corresponding homologous region of the contralateral hemisphere. Identical ROI size and anatomical positioning criteria were applied across animals. ROI definition and image quantification were performed using coded images by investigators blinded to treatment allocation. Areas containing major surface vessels were avoided whenever possible to ensure that the measurements predominantly reflected cortical tissue perfusion rather than signal from large vessels.

Perfusion intensity was obtained from the LSCI software and expressed in arbitrary laser speckle perfusion units (PU). To reduce interanimal variability in absolute signal intensity, cortical perfusion in the ischemic hemisphere was normalized to that of the homologous contralateral hemisphere. Relative cerebral blood flow (rCBF) was calculated as:

rCBF (%) = (ipsilateral MCA-territory perfusion/contralateral homologous perfusion) × 100.

In addition, recovery of ipsilateral perfusion relative to the pre-ischemic state was evaluated by normalizing post-reperfusion measurements to the corresponding baseline value from the same animal. Multiple consecutive images obtained during a stable recording period were averaged to generate a single perfusion value for each animal at each experimental time point, thereby minimizing transient fluctuations and motion-related variability.

The same quantitative analysis pipeline, ROI boundaries, image-display thresholds, and normalization procedures were applied uniformly across all experimental groups. Pseudocolor perfusion maps were used only for representative visualization, whereas group comparisons were performed using individual-animal quantitative rCBF values. Persistent reduction in cortical perfusion after mechanical filament withdrawal was interpreted as post-recanalization tissue hypoperfusion. Because LSCI does not directly visualize capillary obstruction, pericyte constriction, or leukocyte plugging, these measurements were not considered definitive evidence of the cellular mechanisms underlying the no-reflow phenomenon.

### 2.5 Establishment of transient MCAO, mechanical reperfusion, and rt-PA administration

Focal cerebral ischemia was induced using the intraluminal filament method. Rats were anesthetized with isoflurane (RWD Life Science, China, Cat NO. R510-22-10) to maintain an appropriate depth of anesthesia throughout the procedure. The right common carotid artery (CCA), external carotid artery (ECA), and internal carotid artery (ICA) were surgically exposed. The ECA was ligated, and a silicone-coated nylon monofilament (RWD Life Science, Cat NO. MSRC37B200PK10) was inserted into the ICA via the ECA stump until a slight resistance was felt, indicating successful occlusion of the middle cerebral artery origin. Ischemia was maintained for 2 h, after which the filament was withdrawn to restore blood flow. Immediately upon reperfusion, rats designated for thrombolysis received rt-PA (10 mg/kg) via tail vein injection. Owing to the inherent procedural differences between Sham surgery and MCAO induction, complete blinding of the operating surgeon to surgical condition was not feasible. Nevertheless, the surgeon was blinded to the subsequent pharmacological treatment assignment of MCAO animals during model induction and was not involved in behavioral, histological, biochemical, or molecular outcome assessment. It should be noted that reperfusion in this model was achieved mechanically through withdrawal of the intraluminal filament rather than through rt-PA-mediated fibrinolysis. Accordingly, the present experimental paradigm does not directly assess thrombus dissolution or pharmacological recanalization efficacy, but instead evaluates TXL treatment in the setting of rt-PA exposure following mechanically induced reperfusion. Post-surgery, animals were placed in a warming box to maintain body temperature until full recovery from anesthesia. Neurological status was monitored closely within the first 24 h to ensure model stability. Predefined exclusion criteria for MCAO animals included: (1) absence of an evident neurological deficit after recovery from anesthesia, indicating unsuccessful ischemia induction; (2) subarachnoid or intracranial hemorrhage caused by filament insertion; (3) failure to achieve or maintain arterial occlusion or subsequent reperfusion; (4) severe perioperative complications unrelated to the intended ischemic injury; and (5) technical failure that prevented reliable acquisition of the prespecified outcome. Animals that died as a consequence of MCAO were recorded as mortality events rather than being classified as unsuccessful models. All exclusions and deaths were documented before statistical analysis, and no animals were excluded on the basis of treatment response or outcome magnitude.

### 2.6 Neurological function assessment

Neurological deficits were evaluated at 1, 3, 7, and 14 days post-reperfusion by investigators who were blinded to treatment allocation and were not involved in surgery or drug administration. Animals were identified using coded identifiers during behavioral testing, and treatment-group information remained concealed until completion of behavioral scoring and data recording. The mNSS was employed to comprehensively assess motor, sensory, reflex, and balance functions, with scores ranging from 0 (normal) to 18 (maximal deficit); lower scores indicated better functional recovery. Sensorimotor asymmetry was further evaluated using the Corner Turn Test, where rats were placed facing the apex of two boards angled at 30°. The frequency of turns toward the impaired (right) vs. non-impaired (left) side was recorded over 10 trials, and the turning preference ratio was calculated as R/(R+L). Additionally, forelimb use asymmetry was quantified using the Cylinder Test. Rats were placed in a transparent glass cylinder (20 cm diameter, 50 cm height), and their spontaneous vertical exploratory movements were video-recorded for 3 mins. The number of independent touches by the ipsilateral (impaired) forelimb, contralateral (non-impaired) forelimb, and simultaneous bilateral touches were counted. The asymmetry index was calculated as [(Contralateral – Ipsilateral)/Total Touches] × 100%, where a higher value indicated more severe motor coordination impairment.

### 2.7 Evaluation of infarct volume and BBB permeability

To determine infarct volume, brains were rapidly removed at 24 h after reperfusion and sliced into 2-mm thick coronal sections. Sections were stained with 2% 2,3,5-triphenyltetrazolium chloride (TTC; Beyotime, Cat NO. C0652) at 37 °C for 15 mins in the dark, then fixed in 4% paraformaldehyde. Infarct areas were quantified using ImageJ software, and the total infarct volume was calculated with correction for edema. BBB integrity was assessed via Evans Blue (EB) extravasation. Two hours prior to sacrifice, rats received an intravenous injection of 2% EB solution (Beyotime, Cat NO. T3273). Brains were perfused with saline to remove intravascular dye, homogenized in formamide, and incubated at 60 °C. The supernatant was collected, and EB content was quantified spectrophotometrically at 620 nm, expressed as μg/g of brain tissue.

### 2.8 Assessment of BBB tight-junction, basement-membrane, and MMP-9 expression

To further characterize the molecular and structural basis of BBB alterations, ischemic penumbra tissues were collected at 24 h after reperfusion for analysis of tight-junction proteins, basement-membrane components, and matrix metalloproteinase-9 (MMP-9). Protein expression of claudin-5, occludin, zonula occludens-1 (ZO-1), laminin, collagen IV, and MMP-9 was determined by Western blotting. Tissue samples were homogenized in RIPA lysis buffer supplemented with protease and phosphatase inhibitors, and equal amounts of protein were separated by SDS-PAGE and transferred to PVDF membranes. After blocking, membranes were incubated overnight at 4 °C with primary antibodies against claudin-5, occludin, ZO-1, laminin, collagen IV, and MMP-9, followed by the appropriate HRP-conjugated secondary antibodies. Protein bands were visualized using enhanced chemiluminescence and quantified by densitometry. Expression levels were normalized to the corresponding loading control.

In parallel, immunofluorescence staining for claudin-5, ZO-1, and collagen IV was performed on peri-infarct cortical sections to evaluate the continuity and vascular localization of BBB-associated proteins. Images were acquired using identical imaging parameters across groups, and fluorescence intensity and junctional continuity were quantified by investigators blinded to treatment allocation. Because MMP-9 is an important mediator of extracellular-matrix and tight-junction degradation during ischemia/reperfusion injury, particularly in the setting of rt-PA exposure, MMP-9 expression was assessed as an additional index of BBB-disruptive proteolytic activity.

### 2.9 Biochemical assays and ELISA

Ischemic penumbra tissues were homogenized in ice-cold PBS and centrifuged using a high-speed refrigerated centrifuge (Dynamica, Velocity18RPro) to collect supernatants. Levels of pro-inflammatory cytokines, including TNF-α (Beyotime, Cat No. PT516), IL-1β (Beyotime, Cat No. PI303), and IL-6 (Beyotime, Cat No. PI328), were measured using commercial enzyme-linked immunosorbent assay (ELISA) kits according to the manufacturers' protocols. Absorbance was detected using a microplate reader (Thermo Fisher Scientific, Multiskan FC). Oxidative stress markers were also evaluated; superoxide dismutase (SOD) activity (Adison, Cat No. ADS-W-KY011), glutathione peroxidase (GPx) activity (Beyotime, Cat No. S0056), and malondialdehyde (MDA) content (Beyotime, Cat No. S0131S) were determined using colorimetric assay kits, with protein concentrations normalized via the BCA method.

### 2.10 Immunofluorescence staining

Brain tissues were fixed, dehydrated, and embedded in paraffin. Four-micrometer sections were deparaffinized in xylene and rehydrated through a graded ethanol series. Antigen retrieval was performed by heating sections in EDTA buffer (pH 9.0; Wuhan Baitiandu, Cat No. B2002) in a microwave, followed by cooling and washing with PBS. Endogenous peroxidase activity was blocked with 3% H_2_O_2_ for 25 mins. Sections were blocked with 10% normal goat serum (Boster, Cat No. AR1009) for 30 mins and then incubated overnight at 4 °C with primary antibodies against Iba-1 (Proteintech, Cat No. 10904-1-AP) and CD31 (Abcam, Cat No. EPR17259). Following PBS washes, sections were incubated with HRP-conjugated secondary antibodies for 50 mins at room temperature. Signal amplification was achieved using Tyramide Signal Amplification (TSA) with fluorophores CY3 or 488 (Wuhan Baitiandu). For multiplex staining, a second round of microwave antigen retrieval (citrate buffer, pH 6.0), blocking, and antibody incubation was performed. Nuclei were counterstained with DAPI (Wuhan Baitiandu, Cat No. C1030), and sections were mounted with anti-fade mounting medium. Images were captured using a fluorescence microscope.

### 2.11 Immunohistochemistry

Paraffin sections were deparaffinized and subjected to antigen retrieval in citrate buffer (pH 6.0; Wuhan Baitiandu, Cat No. B2010) using microwave heating. After blocking endogenous peroxidase with 3% H_2_O_2_ and non-specific binding with 3% BSA, sections were incubated overnight at 4 °C with anti-CD34 antibody (Abcam, Cat No. EP373Y). Sections were subsequently incubated with HRP-labeled goat anti-rabbit secondary antibody (SeraCare, Cat No. 5220-0336) for 50 mins. Visualization was performed using a DAB kit (Zhongshan Golden Bridge, Cat No. 2005289) under microscopic control, followed by hematoxylin counterstaining, dehydration, and mounting with neutral balsam. Positive staining areas were quantified using image analysis software.

### 2.12 Western blot analysis

Total protein was extracted from ischemic penumbra tissues using RIPA lysis buffer (Beyotime, Cat No. P0013C) supplemented with protease and phosphatase inhibitors (Beyotime, Cat No. P1045). Tissues were homogenized in liquid nitrogen and lysed on ice. Supernatants were collected after centrifugation, and protein concentrations were determined using a BCA assay kit (Epizyme Biotech, Cat No. ZJ101L). Equal amounts of protein (30 μg) were separated by SDS-PAGE using precast gels (Servicebio, Cat No. G2037) and transferred to PVDF membranes (Merck Millipore, Cat No. IPVH00010). Membranes were blocked with 5% non-fat milk and incubated overnight at 4 °C with primary antibodies against VEGF (HUABIO, Cat No. ER30607), BDNF (Proteintech, Cat No. 28205-1-AP), GAP-43 (Proteintech, Cat No. 16971-1-AP), SYN (Proteintech, Cat No. 17785-1-AP), and β-Tubulin (Abclonal, Cat No. A12289). After washing with TBST, membranes were incubated with HRP-conjugated goat anti-rabbit IgG (Abcam, Cat No. ab6721) for 1 h at room temperature. Protein bands were visualized using ECL reagent (Tanon, Cat No. 180-5001) on a chemiluminescence imager (Tanon, Cat# 5200) and quantified by densitometry using ImageJ software.

### 2.13 Golgi-Cox staining

To assess dendritic spine density and neuronal plasticity, fresh brain tissues were processed using a Golgi Stain Kit (Wuhan Baitiandu, Cat# C2144). Tissues were impregnated in Solutions A and B for 2 weeks in the dark (solution refreshed after 6 h), then transferred to Solution C for ≥72 h. Coronal sections (100 μm) were cut on a cryostat (Epredia HM525) at −20 °C to −22 °C and mounted on gelatin-coated slides. Sections were developed in Solutions D and E for 10 min, rinsed, and optionally counterstained with cresyl violet. After dehydration in graded ethanol (50%−100%), clearing in xylene, and resin mounting, dendritic spine density of cortical and hippocampal pyramidal neurons was quantified using a digital slide scanner (3DHISTECH Pannoramic MIDI) and CaseViewer software.

### 2.14 TUNEL assay

Apoptotic cells in paraffin-embedded sections were detected using the TUNEL BrightRed Apoptosis Detection Kit (Beyotime, Cat# C1086). Sections were deparaffinized with an eco-friendly dewaxing solution (Servicebio, Cat# G1128) and subjected to antigen retrieval with Proteinase K (Thermo Fisher, Cat# 4333793) at 37 °C for 20 min. After equilibration, sections were incubated with the TUNEL reaction mixture (containing TdT enzyme and dUTP) at 37 °C for 1 h in a humidified chamber, followed by nuclear counterstaining with DAPI (Beyotime, Cat# C1002) for 10 min. Slides were mounted with anti-fade medium (Thermo Fisher, Cat# P36934), and fluorescence images were acquired to determine the percentage of TUNEL-positive cells.

### 2.15 MMP assay

MMP was evaluated using the JC-1 Assay Kit (Beyotime, Cat# C2005). Fresh frozen brain sections (10 μm) were cut on a cryostat (Thermo Cryotome E), equilibrated to room temperature, and incubated with JC-1 working solution at 37 °C for 1 h in the dark. After PBS washes, nuclei were counterstained with DAPI (Wuhan Baitiandu, Cat# C1030). The red-to-green fluorescence ratio, which reflects high vs. depolarized MMP, was quantified using an inverted fluorescence microscope (Mingmei, MF53). A reduced ratio indicated mitochondrial dysfunction.

For all histological, immunofluorescence, immunohistochemical, biochemical, and molecular outcome assessments, tissue samples, microscopy images, and protein samples were assigned coded identifiers independent of treatment allocation. Quantification of Iba-1, CD31, and CD34 staining, Western blot band densitometry, TUNEL-positive cells, JC-1 red/green fluorescence ratios, and dendritic spine density was performed by investigators blinded to experimental group. The allocation code was not released until completion of image processing, densitometric quantification, and initial statistical analysis.

### 2.16 Statistical analysis

All data are presented as mean ± standard deviation (SD). Statistical analyses were performed using GraphPad Prism version 8.0. Differences between two groups were analyzed using Student's *t*-test, while comparisons among multiple groups were conducted using one-way analysis of variance (ANOVA) followed by Tukey's *post-hoc* test. Behavioral data collected over multiple time points were analyzed using repeated measures ANOVA. A *p*-value of less than 0.05 was considered statistically significant. Statistical analysis was conducted using coded group identifiers whenever feasible, and treatment allocation was disclosed only after completion of prespecified outcome quantification and primary statistical analyses.

## 3 Results

### 3.1 Animal enrollment, model success, and mortality

A total of 65 rats were initially randomized, with 13 animals allocated to each experimental group. All 13 animals in the Sham group completed the experimental procedures. Among the 52 animals subjected to MCAO, three failed to meet the predefined criteria for successful model establishment, corresponding to an unsuccessful-model rate of 5.8% (3/52). These exclusions comprised one animal in the MCAO group, one in the MCAO + rt-PA group, and one in the MCAO + rt-PA + TXL-Low group. Among the remaining 49 successfully modeled animals, three died during the postoperative follow-up period, corresponding to a mortality rate of 6.1% among successfully modeled MCAO animals and 4.6% of all initially enrolled animals. One death occurred in the MCAO group, one in the MCAO + rt-PA group, and one in the MCAO + rt-PA + TXL-High group. Consequently, 59 animals were available for the final analyses: Sham, *n* = 13; MCAO, *n* = 11; MCAO + rt-PA, *n* = 11; MCAO + rt-PA + TXL-Low, *n* = 12; and MCAO + rt-PA + TXL-High, *n* = 12. No additional animals were excluded because of the magnitude or direction of the observed treatment response. Mortality was recorded throughout the experimental period and was reported separately for each treatment group together with unsuccessful model induction and other predefined exclusions. Because systematic post-mortem determination of the cause of death was not performed, mortality events could not be reliably classified as hemorrhagic or non-hemorrhagic in origin.

### 3.2 TXL improves neurological recovery and reduces tissue injury in rt-PA-exposed MCAO rats

Behavioral assessments revealed that rats in the MCAO group exhibited severe neurological deficits and impaired motor coordination compared to the Sham group. Treatment with rt-PA alone provided modest improvement, whereas administration of TXL in rt-PA-treated animals was associated with additional, dose-dependent amelioration of neurological deficits. This comparison demonstrates an incremental treatment benefit in the rt-PA-treated setting but, in the absence of a TXL-alone group, does not establish pharmacological additivity or synergy between TXL and rt-PA. To elucidate the mechanisms by which TXL preserves the NVU, we evaluated its effects on inflammatory responses and vascular remodeling. Neurological function was assessed using the mNSS, corner turn test, and cylinder test. MCAO produced marked neurological deficits, as reflected by persistently elevated mNSS values compared with the Sham group. rt-PA treatment improved neurological recovery, whereas the addition of TXL produced a further reduction in mNSS, with the greatest improvement observed in the high-dose TXL group over the 14-day observation period (Figure 1A). Consistent with these findings, MCAO markedly increased the percentage of turns toward the impaired side in the corner test and increased forelimb-use asymmetry in the cylinder test. These abnormalities were partially attenuated by rt-PA and were further reduced by TXL treatment, with the high-dose TXL group showing the greatest improvement among the MCAO-treated groups (Figures 1B, C). BBB integrity was subsequently evaluated using Evans Blue extravasation. Representative brain sections showed pronounced Evans Blue leakage after MCAO, indicating substantial disruption of the blood–brain barrier. rt-PA treatment attenuated this leakage, whereas administration of TXL in rt-PA-treated animals was associated with a further reduction in Evans Blue extravasation, with the high-dose TXL group showing the least apparent leakage among the ischemic treatment groups (Figure 1D). Cerebral infarction was assessed by TTC staining at 24 h after reperfusion. The MCAO group exhibited extensive pale infarcted regions, whereas rt-PA treatment reduced the infarcted area. Addition of TXL further decreased infarct size in a dose-dependent manner, with the high-dose TXL group exhibiting the smallest infarcted area among the MCAO-treated groups (Figure 1E). Quantitative analysis confirmed a significant reduction in infarcted area following rt-PA treatment and a further reduction after low- and high-dose TXL administration, with the greatest reduction observed in the high-dose TXL group (Figure 1F). LSCI was used to assess cortical cerebral perfusion. MCAO caused a marked reduction in blood flow within the ipsilateral MCA territory compared with the Sham group. Following reperfusion, cortical perfusion partially recovered but remained impaired in the MCAO and MCAO + rt-PA groups. TXL administration was associated with a further improvement in ipsilateral cortical perfusion, with a greater recovery observed in the high-dose TXL group (Figure 1G). Relative ipsilateral cerebral blood flow was quantified for each animal as the ratio of the ipsilateral ROI to the corresponding contralateral ROI × 100%. Quantitative analysis confirmed markedly reduced relative cerebral blood flow after MCAO, partial restoration following rt-PA treatment, and further increases following low- and high-dose TXL treatment, with the high-dose group showing the greatest perfusion recovery and approaching the level observed in Sham animals (Figure 1H).

**Figure 1:**
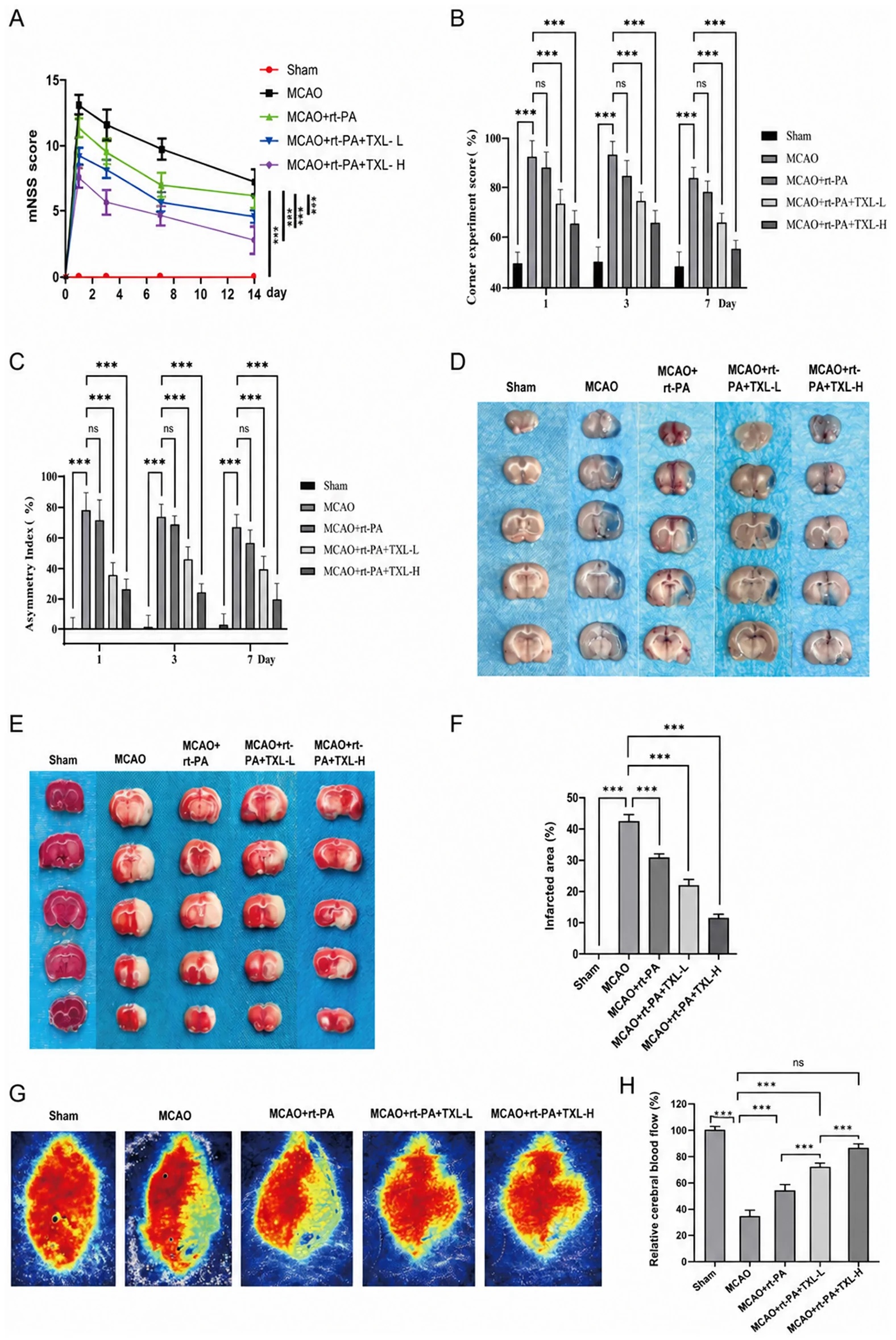
TXL combined with rt-PA improves neurological recovery, blood–brain barrier integrity, cerebral infarction, and cortical perfusion after MCAO. **(A)** Time-course changes in modified neurological severity score (mNSS) from day 1 to day 14 after reperfusion. Lower scores indicate better neurological function. **(B)** Corner turn test results at days 1, 3, and 7 after reperfusion. The percentage of turns toward the impaired side was recorded over 10 trials; higher values indicate greater sensorimotor asymmetry. **(C)** Cylinder test results at days 1, 3, and 7 after reperfusion. The asymmetry index was calculated as [(Contralateral – Ipsilateral)/Total Touches] × 100%; higher values indicate greater forelimb-use asymmetry. **(D)** Representative Evans Blue-stained brain sections showing blood–brain barrier permeability at 24 h after reperfusion. Blue staining indicates extravasation of Evans Blue into the brain parenchyma. **(E)** Representative TTC-stained brain sections showing cerebral infarction at 24 h after reperfusion. Pale areas represent infarcted tissue. Scale bar: 1 cm. **(F)** Quantification of infarcted area based on TTC staining. **(G)** Representative laser speckle contrast imaging (LSCI) maps of cortical cerebral blood flow following MCAO and treatment with rt-PA and/or TXL. Red/yellow regions indicate relatively higher perfusion, whereas blue regions indicate reduced perfusion. **(H)** Quantification of relative ipsilateral cerebral blood flow. LSCI, laser speckle contrast imaging, mNSS, modified neurological severity score; MCAO, middle cerebral artery occlusion; rt-PA, recombinant tissue plasminogen activator; TXL, Tongxinluo capsule. ^***^P < 0.001. ns, not significant.

### 3.3 TXL is associated with reduced neuroinflammation and enhanced vascular-related responses following I/R injury

To further investigate the mechanisms underlying the protective effects of TXL on the neurovascular unit, inflammatory responses and vascular-associated changes in the ischemic brain were evaluated. ELISA of the ischemic penumbra demonstrated a marked increase in the concentrations of the pro-inflammatory cytokines TNF-α, IL-1β, and IL-6 after MCAO compared with the Sham group. rt-PA treatment significantly reduced the levels of all three inflammatory mediators. Addition of TXL to rt-PA treatment produced a further reduction in TNF-α, IL-1β, and IL-6, with the greatest decreases observed in the high-dose TXL group (Figures 2A–C). The concurrent reduction in TNF-α, IL-1β, and IL-6 levels indicates attenuation of the inflammatory environment after TXL administration; however, the present analyses do not determine whether this effect results from altered microglial polarization, reduced microglial activation intensity, modulation of infiltrating immune cells, or other inflammatory regulatory mechanisms. These findings indicate that TXL treatment was associated with an additional attenuation of the post-ischemic inflammatory response beyond that observed with rt-PA alone.

**Figure 2:**
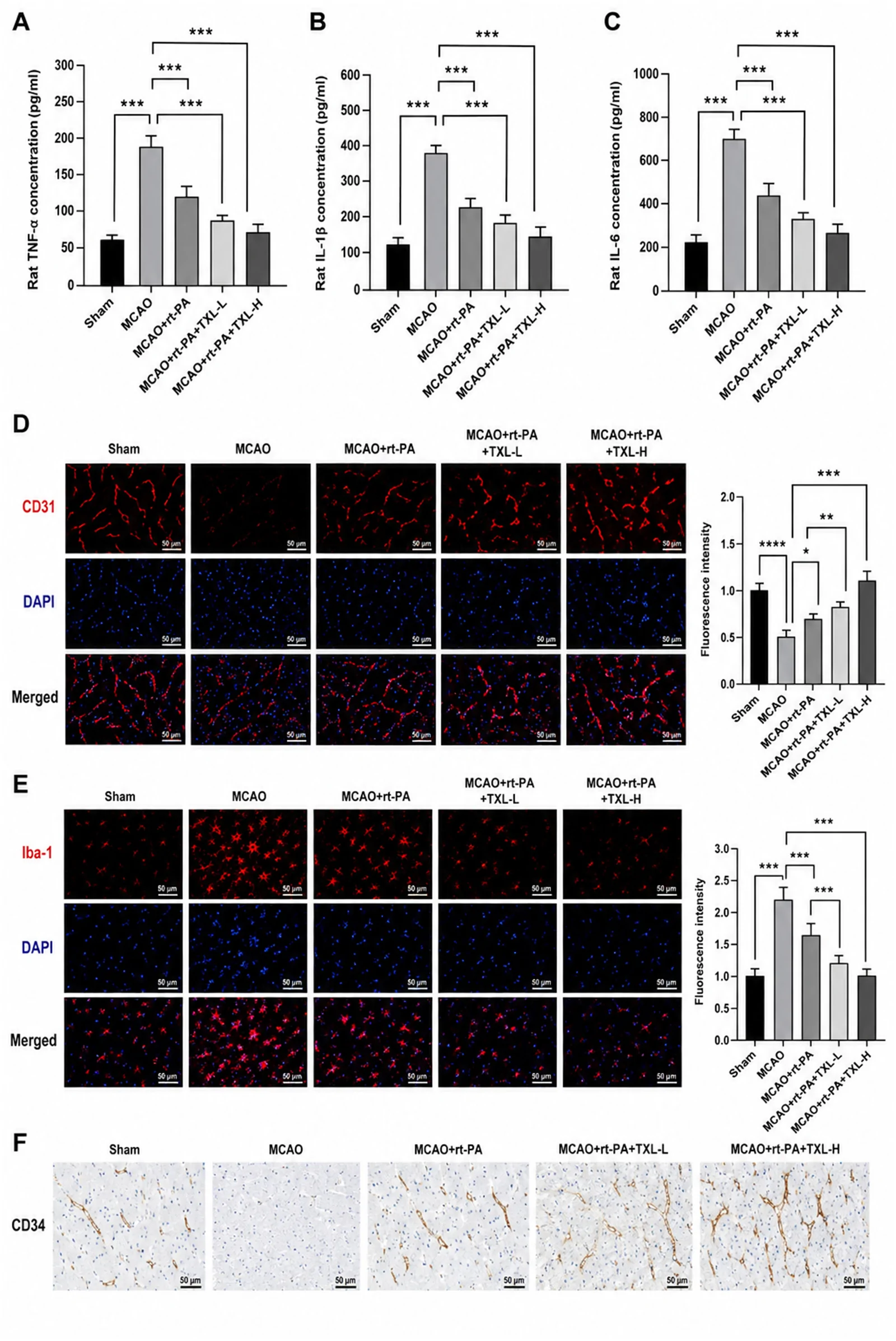
**(A)** Quantification of TNF-α protein levels in the ischemic penumbra. **(B)** Quantification of IL-1β protein levels in the ischemic penumbra. **(C)** Quantification of IL-6 protein levels in the ischemic penumbra. **(D)** Immunofluorescence staining for CD31 (red, endothelial cell marker) and DAPI (blue, nuclei) in the ischemic boundary zone, with corresponding quantitative analysis of fluorescence intensity. Scale bar: 50 μm. **(E)** Immunofluorescence staining for Iba-1 (red, microglial marker) and DAPI (blue, nuclei) in the ischemic penumbra, with corresponding quantitative analysis of fluorescence intensity. Scale bar: 50 μm. **(F)** Immunohistochemical staining for CD34 in the ischemic boundary zone. Scale bar: 50 μm. *P < 0.05, **P < 0.01, and ***P < 0.001. DAPI, 4′,6-diamidino-2-phenylindole; Iba-1, ionized calcium-binding adaptor molecule 1; MCAO, middle cerebral artery occlusion; rt-PA, recombinant tissue plasminogen activator; TXL, Tongxinluo.

Consistent with a protective effect on the cerebral microvasculature, CD31 immunofluorescence revealed marked alterations in endothelial-associated vascular structures after MCAO. The MCAO group exhibited substantially weaker CD31 fluorescence than the Sham group, whereas rt-PA treatment partially restored CD31 staining. Both TXL-treated groups showed a further increase in CD31-positive vascular structures and fluorescence intensity, with the high-dose TXL group exhibiting the greatest recovery (Figure 2D). Quantitative fluorescence analysis was consistent with the representative images, showing significantly reduced CD31 intensity after MCAO and progressive restoration following rt-PA, low-dose TXL, and high-dose TXL treatment.

Microglial activation was assessed by Iba-1 immunofluorescence. MCAO resulted in a pronounced increase in Iba-1-positive cells and fluorescence intensity in the ischemic penumbra compared with the Sham group, consistent with robust post-ischemic microglial activation. rt-PA treatment reduced Iba-1 fluorescence, and the addition of TXL produced a further reduction, with the lowest intensity observed in the high-dose TXL group (Figure 2E). Quantitative analysis confirmed the progressive decrease in Iba-1 fluorescence across the rt-PA and TXL treatment groups, indicating reduced microglia-associated activation following treatment. However, because Iba-1 is a general marker of microglial/macrophage activation and does not distinguish between pro-inflammatory and reparative phenotypes, these findings should not be interpreted as direct evidence of selective suppression of pro-inflammatory microglial polarization.

Immunohistochemical staining for CD34 was further used to examine vascular-associated remodeling in the ischemic boundary zone. Representative sections showed sparse CD34-positive vascular structures after MCAO, whereas greater CD34-positive staining was observed following rt-PA treatment. The TXL-treated groups exhibited a further apparent increase in CD34-positive vessel-like structures, particularly in the high-dose group (Figure 2F). These observations are consistent with enhanced vascular-associated remodeling after TXL treatment. However, because Figure 2F provides representative immunohistochemical images without corresponding quantitative analysis, the CD34 findings should be interpreted qualitatively and should not be used alone to establish increased angiogenesis, functional vessel formation, or restoration of microvascular perfusion. However, the present experiments do not establish whether these effects directly restore capillary-level perfusion or alleviate the cellular and intravascular components of no-reflow. Collectively, these findings demonstrate that adjunctive TXL treatment was associated with reduced inflammatory cytokine levels and Iba-1 immunoreactivity, together with increased CD31-positive microvascular density and CD34-positive lumen-like structures. These concurrent alterations are consistent with a more favorable inflammatory and vascular response following ischemia/reperfusion injury. However, because microglial and vascular responses were assessed in parallel rather than experimentally manipulated, the present findings do not establish that suppression of microglial activation directly caused the observed vascular changes or that the CD31/CD34-positive structures represented fully mature and functionally integrated neovasculature. Furthermore, CD34 immunoreactivity primarily reflects the presence of endothelial-associated structures and is not sufficient to confirm complete vascular maturation. Therefore, the CD34-positive lumen-like structures observed in the present study should be interpreted as evidence of vascular-associated remodeling rather than definitive proof of mature, functionally integrated neovascularization. Additional analyses using markers of vessel maturation, such as pericyte-associated proteins (e.g., α-SMA or NG2) and endothelial junctional maturation markers (e.g., VE-cadherin or vWF), will be required to further characterize vascular stability and maturation.

### 3.4 TXL is associated with reduced oxidative stress and increased antioxidant enzyme activity

Oxidative stress markers were assessed to determine the antioxidant potential of TXL. MCAO induction led to a significant elevation in malondialdehyde (MDA) levels, indicative of severe lipid peroxidation, alongside a marked decrease in the activities of SOD and glutathione peroxidase (GPx). TXL treatment significantly reduced MDA content and increased SOD and GPx activities compared with the MCAO and MCAO + rt-PA groups (Figures 3A–C). These findings indicate that TXL treatment was associated with reduced lipid peroxidation and preservation of endogenous antioxidant enzyme activity following ischemia/reperfusion injury. However, because the present experiments did not directly assess upstream redox-sensitive signaling molecules or cellular ROS production, these biochemical changes do not identify the molecular pathway responsible for the antioxidant effect of TXL.

**Figure 3:**
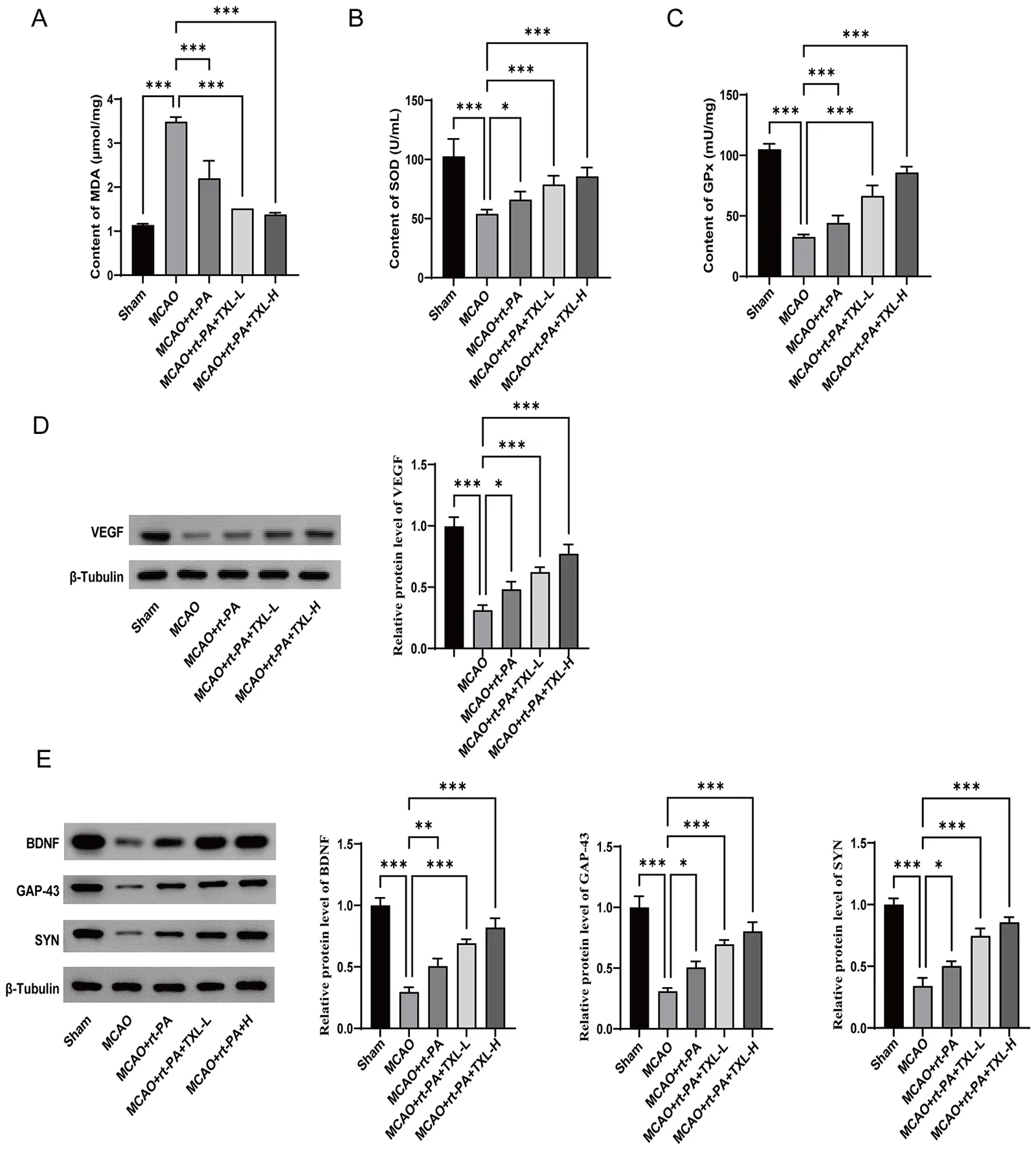
**(A)** Malondialdehyde (MDA) content in the ischemic penumbra, measured by spectrophotometry. **(B)** Superoxide dismutase (SOD) activity in the ischemic penumbra, assessed by spectrophotometric assay. **(C)** Glutathione peroxidase (GPx) activity in the ischemic penumbra, assessed by spectrophotometric assay. **(D)** Western blot analysis of vascular endothelial growth factor (VEGF) protein expression in the ischemic penumbra. **(E)** Western blot analysis of brain-derived neurotrophic factor (BDNF), growth-associated protein 43 (GAP-43), and synapsin I (SYN) in the ischemic penumbra. MDA, malondialdehyde; SOD, superoxide dismutase; GPx, glutathione peroxidase; VEGF, vascular endothelial growth factor; BDNF, brain-derived neurotrophic factor; GAP-43, growth-associated protein 43; SYN, synapsin I; β-Tubulin, beta-tubulin. ^*^P < 0.05, ^**^P < 0.01, and ^***^P < 0.001.

### 3.5 TXL upregulates expression of proteins associated with neural repair and synaptic plasticity

Western blot analysis was performed to evaluate changes in proteins associated with vascular repair and neuronal plasticity following TXL treatment. Expression of VEGF, BDNF, GAP-43, and Synapsin I was quantified as molecular correlates of vascular- and neural plasticity-related responses; however, these proteins were not considered as direct measures of functional synaptic connectivity or neural circuit reconstruction. The results showed that TXL treatment significantly upregulated the protein expression of VEGF, providing a molecular basis for the observed angiogenesis described above (Figure 3D). Furthermore, TXL treatment increased the expression of BDNF, GAP-43, and Synapsin I, proteins associated with neuronal survival, neurite growth, and synaptic plasticity. The coordinated elevation of these proteins is consistent with a molecular environment favorable to post-ischemic neural plasticity. However, because these markers do not directly quantify synapse number, synaptic organization, or electrophysiological function, the present findings should not be interpreted as direct evidence of synaptic reconstruction or restoration of functional neural circuits (Figure 3E).

### 3.6 TXL preserves MMP, reduces apoptotic cell labeling, and increases dendritic spine density

Finally, we assessed the effects of TXL on mitochondrial health and neuronal apoptosis. TUNEL assay revealed a high proportion of apoptotic cells in the ischemic penumbra of the MCAO and MCAO + rt-PA groups. TXL treatment significantly reduced the number of TUNEL-positive cells, demonstrating a potent anti-apoptotic effect (Figure 4A). JC-1 staining demonstrated a marked reduction in MMP, reflected by a decreased red/green fluorescence ratio following MCAO. TXL administration significantly increased the JC-1 red/green fluorescence ratio, indicating partial preservation of MMP. Because JC-1 provides an indirect measure of mitochondrial membrane polarization, these findings should not be interpreted as comprehensive evidence of restored mitochondrial bioenergetic function (Figure 4B). To evaluate structural synaptic recovery, Golgi-Cox staining was employed. Quantitative analysis showed that MCAO led to a significant loss of dendritic spines on pyramidal neurons. TXL treatment, particularly at the high dose, significantly increased dendritic spine density compared with the model group, indicating preservation or recovery of a morphological feature associated with neuronal plasticity. However, increased spine density alone does not establish that these structures formed mature functional synapses or restored synaptic transmission (Figure 4C). Collectively, TXL treatment was associated with preservation of MMP, reduced TUNEL-positive cell labeling, and increased dendritic spine density. However, these parallel observations do not establish that preservation of MMP directly caused the reduction in apoptosis or subsequent structural plasticity.

**Figure 4:**
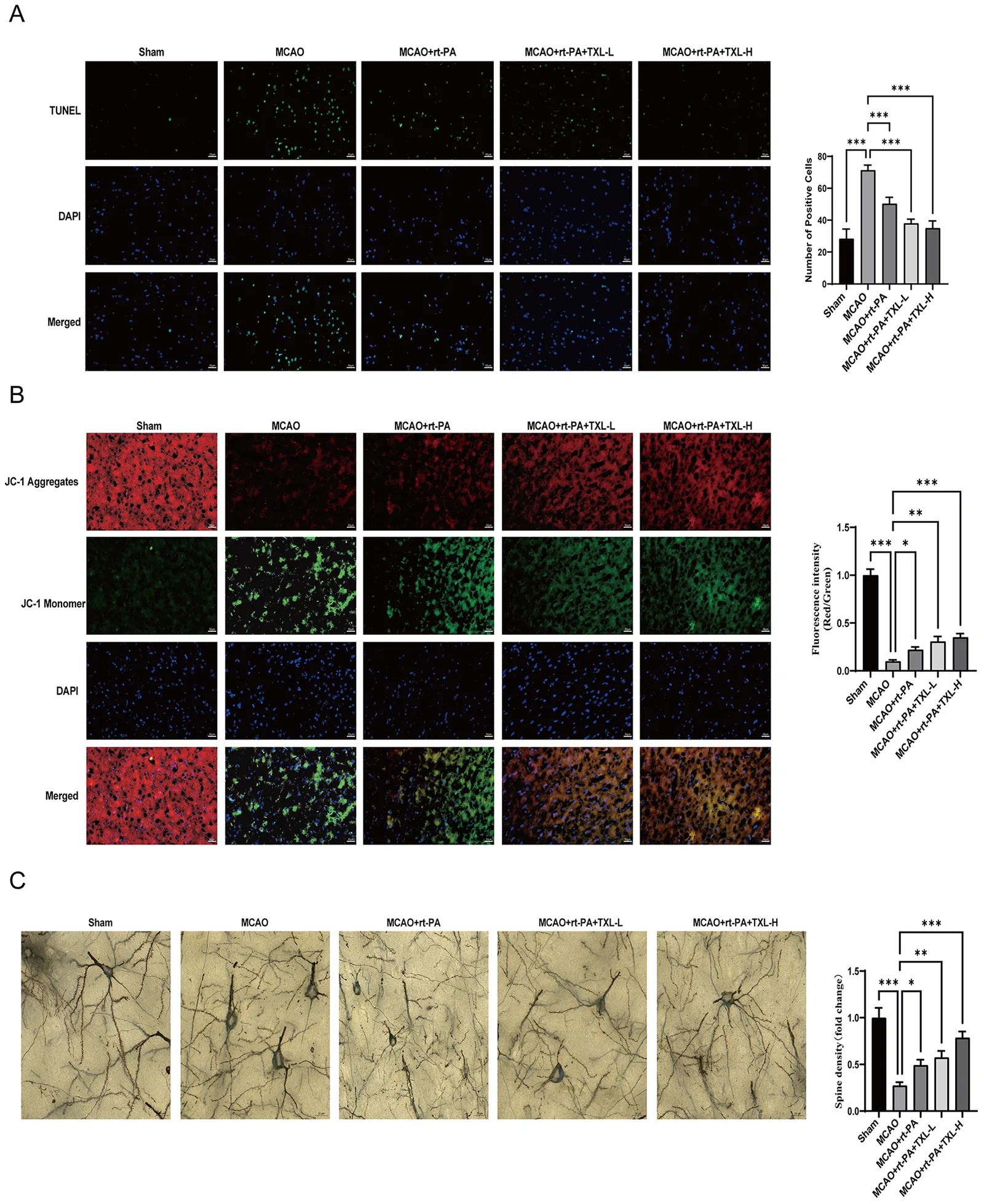
**(A)** TUNEL staining for detection of apoptotic cells in the ischemic penumbra. Scale bar: 50 μm. **(B)** JC-1 staining to assess mitochondrial membrane potential (MMP). Scale bar: 50 μm. **(C)** Golgi-Cox staining of pyramidal neurons in the cortex or hippocampus. Scale bar: 20 μm. TUNEL, terminal deoxynucleotidyl transferase dUTP nick end labeling; MMP, mitochondrial membrane potential; MCAO, middle cerebral artery occlusion; rt-PA, recombinant tissue plasminogen activator; TXL, Tongxinluo capsule. ^*^P < 0.05, ^**^P < 0.01, and ^***^P < 0.001.

## 4 Discussion

Rt-PA remains the primary pharmacological intervention for acute ischemic stroke, yet its clinical utility is frequently constrained by incomplete microvascular reperfusion and secondary reperfusion injury (24, 25). In the present study, adjunctive TXL treatment was associated with improved neurological outcomes, enhanced cortical perfusion, reduced BBB leakage, attenuation of inflammatory and oxidative-stress responses, increased expression of vascular- and plasticity-related markers, preservation of MMP, reduced apoptotic cell labeling, and increased dendritic spine density in rt-PA-treated MCAO rats. Considered collectively, these observations suggest that TXL influences multiple biological processes relevant to NVU integrity and post-ischemic recovery. However, because these responses were assessed predominantly through parallel behavioral, hemodynamic, histological, biochemical, and molecular measurements, the present data do not establish that TXL directly orchestrates comprehensive remodeling of the entire NVU, nor do they demonstrate a causal hierarchy linking microglial suppression, vascular responses, mitochondrial preservation, and synaptic plasticity.

A pivotal finding in our work is the dose-dependent restoration of cortical cerebral blood flow observed via laser speckle contrast imaging. While rt-PA effectively dissolves macroscopic thrombi, it often fails to resolve microvascular obstructions caused by endothelial swelling, pericyte contraction, and leukocyte plugging (26). However, the present filament MCAO model does not contain an intravascular thrombus requiring rt-PA-mediated fibrinolysis. Therefore, the effects of TXL cannot be interpreted as enhancement of rt-PA thrombolytic activity or vessel recanalization. This discrepancy explains why recanalization of large vessels does not always translate into tissue salvage. Our quantitative LSCI findings indicate that TXL treatment was associated with improved cortical tissue perfusion following reperfusion. This improvement may reflect attenuation of post-recanalization microvascular dysfunction; however, the present study did not directly assess the defining capillary-level components of no-reflow, including microvascular obstruction, endothelial swelling, pericyte constriction, leukocyte adhesion or plugging, and platelet/fibrin-rich microthrombi. Moreover, although reduced Evans Blue extravasation and increased CD31/CD34-associated vascular signals are compatible with improved vascular integrity and remodeling, these measurements do not directly demonstrate restoration of functional capillary patency. Accordingly, the present findings support a beneficial effect of TXL on post-reperfusion cortical perfusion but do not establish a specific mechanism of no-reflow reversal.

The inflammatory response following reperfusion plays a complex role in stroke pathophysiology (27, 28). While initial immune activation aids in debris clearance, excessive microglial activation releases cytotoxic cytokines such as TNF-α, IL-1β, and IL-6, which damage neurons and degrade the extracellular matrix required for vascular reconstruction (29, 30). The results indicated that TXL treatment was associated with reduced microglia-associated inflammatory responses, as reflected by decreased Iba-1 immunoreactivity and lower levels of inflammatory cytokines. However, because Iba-1 is not a phenotype-specific marker, these findings do not establish that TXL selectively inhibits pro-inflammatory microglial polarization or promotes a reparative microglial phenotype. The concomitant reduction in inflammatory markers and increase in vascular-associated markers raises the possibility that attenuation of the inflammatory environment may favor vascular repair; however, a direct causal relationship between these processes was not established in the present study. In a highly inflammatory milieu, newly formed vessels are often leaky and dysfunctional. Conversely, the low-inflammatory environment associated with TXL treatment was accompanied by increased vascular-associated signals, including CD31-positive microvascular structures and CD34-positive lumen-like staining patterns. These findings are consistent with enhanced vascular remodeling; however, they do not by themselves establish formation of fully mature or functionally integrated vascular networks. These parallel findings are consistent with coordinated modulation of inflammatory and vascular responses, although whether suppression of inflammation directly facilitates vascular maturation remains to be determined, ensuring adequate delivery of oxygen and nutrients to the ischemic penumbra.

Beyond vascular protection, our study reveals that TXL exerts profound effects on neuronal survival and circuit reconstruction at the subcellular level. I/R injury triggers mitochondrial dysfunction, leading to energy failure and the initiation of intrinsic apoptotic pathways (31, 32). We observed that TXL treatment significantly restored MMP and reduced the number of TUNEL-positive apoptotic cells. This preservation of mitochondrial health is critical for maintaining the high energy demands of synaptic transmission. Consistent with the molecular findings, Golgi-Cox staining demonstrated increased dendritic spine density following TXL treatment, providing a structural correlate of enhanced neuronal plasticity. The concomitant increases in BDNF, GAP-43, and Synapsin I further indicate activation of molecular processes associated with neuronal survival, neurite growth, and synaptic plasticity (33–35). Nevertheless, neither these protein changes nor dendritic spine density directly demonstrates formation of mature synapses, restoration of synaptic transmission, or reconstruction of functional neural circuits. Accordingly, the observed behavioral improvement may be associated with enhanced neural plasticity, but a direct mechanistic link between these molecular and structural alterations and functional circuit recovery remains to be established.

The antioxidant capacity of TXL also contributes significantly to its neuroprotective profile. Reperfusion leads to a burst of reactive oxygen species that cause lipid peroxidation and further cellular damage (36). Our biochemical analyses demonstrated that TXL treatment was associated with reduced MDA accumulation and increased SOD and GPx activities, consistent with attenuation of lipid peroxidation and preservation of endogenous antioxidant capacity following ischemia/reperfusion injury. One potential mechanism that warrants investigation is activation of the Nrf2/HO-1 antioxidant-response pathway. Under oxidative stress, Nrf2-dependent transcription can promote expression of cytoprotective and antioxidant proteins, thereby enhancing cellular resistance to reactive oxygen species. However, Nrf2 activation, nuclear translocation, HO-1 expression, and downstream antioxidant-response elements were not directly evaluated in the present study. Therefore, the current findings demonstrate an antioxidant-associated effect of TXL but do not establish Nrf2/HO-1 or any other specific redox pathway as the causal mechanism. The concurrent reduction in oxidative-stress markers and preservation of MMP indicates a potential relationship between redox regulation and mitochondrial status (37, 38). However, mitochondrial ROS production was not directly quantified, and the present data therefore do not establish a reciprocal feedback mechanism between oxidative stress and mitochondrial function.

Several strengths distinguish this study. First, we utilized a clinically relevant model combining rt-PA with adjunctive therapy, mirroring real-world treatment scenarios. Second, the application of LSCI enabled dynamic assessment and quantitative comparison of cortical tissue perfusion, providing complementary hemodynamic information beyond that obtainable from static histological measurements. Nevertheless, LSCI does not directly resolve individual capillary-level mechanisms underlying impaired reperfusion. Third, our comprehensive analysis spanning behavioral, hemodynamic, histological, and molecular levels offers a holistic understanding of how TXL orchestrates NVU remodeling. The observation of greater treatment effects at 0.6 g/kg than at 0.3 g/kg supports a dose-responsive association within the dose range examined. However, because only two TXL doses were evaluated and pharmacokinetic exposure was not directly measured, the present study cannot define the complete dose-response relationship or identify an optimal therapeutic dose.

However, certain limitations must be acknowledged. Although we identified key protein markers involved in angiogenesis and synaptic plasticity, the specific upstream signaling pathways mediating these effects remain to be fully elucidated. Future studies employing pathway inhibitors or genetic models are needed to determine whether TXL acts through canonical routes, such as PI3K/AKT, Nrf2/HO-1, or NF-κB. Additionally, while our current data demonstrate strong efficacy in reducing infarct volume and improving short-term function, the long-term stability of the newly formed vascular networks and synaptic connections requires further investigation. Extended follow-up periods would clarify whether the promoted vascular and synaptic changes translate into sustained functional recovery without risks of aberrant growth. An additional important limitation is that the present study was conducted exclusively in adult male rats. Biological sex is an important determinant of ischemic stroke pathophysiology and may influence neuroinflammatory responses, blood–brain barrier integrity, vascular injury, and responses to reperfusion-related interventions. Consequently, the present findings cannot be assumed to generalize directly to females, and the magnitude or mechanisms of TXL-associated protection may differ according to sex. Future studies should therefore incorporate both male and female animals, with adequately powered sex-stratified analyses, to determine whether the observed effects are reproducible across sexes and whether biological sex modifies the response to TXL in the rt-PA-treated ischemia/reperfusion setting. An additional limitation is that the present study was not designed to establish causal relationships among the individual NVU-related responses associated with TXL treatment. Although reduced microglial activation and inflammatory mediator levels occurred concomitantly with increased vascular-associated markers, preservation of MMP, reduced apoptosis, and enhancement of synaptic plasticity-related indices, these findings were derived primarily from parallel biomarker and histological assessments. Cell-specific depletion approaches, pathway-selective pharmacological inhibitors, genetic manipulation, and formal mediation experiments were not performed. Consequently, it remains uncertain whether these responses constitute sequentially interconnected components of a unified TXL-mediated mechanism or represent partially independent effects of treatment. Another limitation is that vascular maturation was inferred primarily from CD31 and CD34-associated staining patterns. Although these markers provide information regarding endothelial-associated structures, they do not directly evaluate vessel stabilization, pericyte recruitment, endothelial junctional integrity, or functional vascular integration. Future studies incorporating pericyte coverage markers, including α-SMA or NG2, together with endothelial maturation markers such as VE-cadherin or vWF, would provide a more comprehensive evaluation of vascular maturation following TXL treatment. Moreover, increased CD31/CD34 staining cannot independently establish complete vascular maturation or functional integration, while increased BDNF, GAP-43, and Synapsin I expression should be interpreted as molecular correlates of neural plasticity rather than direct evidence of neural circuit reconstruction. Future studies incorporating cell-specific and pathway-specific interventions are required to determine the causal relationships among inflammatory regulation, vascular remodeling, mitochondrial preservation, and neural plasticity. Another limitation is that the present study did not directly characterize capillary-level mechanisms underlying post-recanalization hypoperfusion. Although LSCI demonstrated improved cortical perfusion and histological analyses revealed favorable vascular-associated changes, no direct measurements of capillary patency, red blood cell transit, endothelial swelling, pericyte constriction, leukocyte adhesion or plugging, or platelet/fibrin deposition were performed. Consequently, the data cannot determine which specific microvascular processes account for the observed perfusion improvement or establish that TXL directly prevents the no-reflow phenomenon. Future studies should incorporate *in vivo* microvascular perfusion labeling, high-resolution capillary imaging, and cellular or intravascular markers of microvascular obstruction to clarify the underlying mechanism. An additional limitation relates to the experimental stroke model employed. In the present intraluminal filament MCAO model, cerebral reperfusion was initiated mechanically by withdrawal of the occluding filament before rt-PA administration. Importantly, the present model evaluated TXL administration in the context of mechanically induced reperfusion followed by rt-PA exposure. Because no intravascular thrombus was present, the study was not designed to assess enhancement of rt-PA-mediated fibrinolysis, thrombus dissolution, or pharmacological recanalization. Future studies using thromboembolic stroke models with direct assessment of thrombus resolution and vessel recanalization are required to determine whether TXL influences thrombolytic efficacy. A further limitation is that microglial responses were characterized primarily by Iba-1 immunoreactivity together with measurements of pro-inflammatory cytokines. Although these findings indicate an alteration in microglial activation and the inflammatory milieu, Iba-1 is a general marker of microglial/macrophage activation and does not distinguish between pro-inflammatory and reparative microglial phenotypes or define the specific intracellular pathways involved. Therefore, the present data cannot establish whether TXL selectively suppresses a pro-inflammatory microglial state or promotes a reparative phenotype. Future studies incorporating phenotype-specific markers, such as CD86, iNOS, NLRP3, and NF-κB pathway components to evaluate pro-inflammatory responses, along with CD206 and Arg1 to assess reparative-associated responses, would provide a more precise characterization of TXL-mediated immunomodulation. Future studies should incorporate a broader panel of phenotype- and pathway-associated markers, including CD68, iNOS, CD86, NLRP3, and NF-κB p65 for pro-inflammatory activation and Arg1 and CD206 for reparative-associated responses. Moreover, the use of microglia-specific markers, such as TMEM119 or P2RY12 in combination with cell-specific co-localization analyses within the ischemic penumbra would help distinguish resident microglial responses from infiltrating myeloid cells and provide a more precise characterization of TXL-associated immunomodulatory effects. An important limitation with direct translational relevance is the absence of a systematic assessment of thrombolysis-associated safety. Although BBB permeability was evaluated using Evans Blue extravasation, the present study did not prospectively quantify hemorrhagic transformation, intracerebral hemorrhage, brain hemoglobin content, systemic bleeding events, or coagulation parameters. Cerebral edema was also not evaluated as an independent outcome, although edema correction was applied during TTC-based infarct-volume calculation. Consequently, the current experiments cannot establish the hemorrhagic or coagulation-related safety of TXL when administered in the setting of rt-PA exposure. This limitation is particularly important because any treatment proposed for use alongside thrombolysis must demonstrate that potential neuroprotective benefits are not accompanied by an increased risk of intracranial or systemic bleeding. Future studies should therefore incorporate prospectively defined safety endpoints, including standardized hemorrhagic-transformation grading, quantitative brain hemoglobin measurement, gross and histological assessment of intracerebral hemorrhage, cerebral water content or hemispheric swelling, treatment-specific mortality with determination of cause of death, and systemic coagulation indices such as prothrombin time, activated partial thromboplastin time, fibrinogen concentration, and platelet count. Evaluation in a thromboembolic stroke model would also be important to determine the overall benefit–risk profile of TXL when administered with pharmacologically active rt-PA-mediated thrombolysis. Finally, although TXL showed a favorable safety profile under the present experimental conditions, comprehensive evaluation of its efficacy and safety across both sexes, as well as in models incorporating larger infarcts, advanced age, and clinically relevant comorbidities, will be necessary before translational conclusions can be drawn. In particular, future studies should determine whether sex modifies the effects of TXL on reperfusion-associated injury, BBB integrity, neuroinflammation, and neurological recovery.

## 5 Conclusion

In conclusion, adjunctive TXL treatment was associated with improved neurological outcomes, cortical perfusion, BBB integrity, and reduced infarct-related injury in rt-PA-treated MCAO rats. These outcomes were accompanied by reduced neuroinflammatory and oxidative-stress markers, increased vascular-associated parameters, preservation of MMP, decreased apoptotic labeling, and enhanced molecular and structural indices of neuronal plasticity. Collectively, these findings suggest that TXL may exert multimodal protective effects across several biological processes relevant to NVU integrity and post-ischemic recovery. However, the present findings do not establish that TXL directly orchestrates comprehensive NVU remodeling or that microglial suppression, vascular responses, mitochondrial preservation, and synaptic plasticity constitute a causally interconnected pathway. Mechanistic studies employing pathway-specific inhibition, cell-specific manipulation, and longitudinal assessment of vascular and synaptic functionality are required to determine how these responses are functionally related. Accordingly, the present results support further preclinical investigation of TXL as an adjunctive neuroprotective strategy in the setting of rt-PA treatment, while additional thromboembolic models are required to determine whether TXL affects rt-PA-mediated thrombolysis or recanalization.

## Data Availability

The datasets presented in this study can be found in online repositories. The names of the repository/repositories and accession number(s) can be found in the article/supplementary material.
